# Thermal activation energy of 3D vortex matter in NaFe_1−*x*_Co_*x*_As (*x* = 0.01, 0.03 and 0.07) single crystals

**DOI:** 10.1038/s41598-017-11371-1

**Published:** 2017-09-07

**Authors:** W. J. Choi, Y. I. Seo, D. Ahmad, Yong Seung Kwon

**Affiliations:** Department of Emerging Materials Science, DGIST, Daegu, 42988 Republic of Korea

## Abstract

We report on the thermally activated flux flow dependency on the doping dependent mixed state in NaFe_1−*x*_Co_*x*_As (*x* = 0.01, 0.03, and 0.07) crystals using the magnetoresistivity in the case of *B*//*c*-axis and *B*//*ab*-plane. It was found clearly that irrespective of the doping ratio, magnetoresistivity showed a distinct tail just above the *T*_c,offset_ associated with the thermally activated flux flow (TAFF) in our crystals. Furthermore, in TAFF region the temperature dependence of the activation energy follows the relation $U(T,B)={U}_{0}(B){(1-T/{T}_{c})}^{q}$ with *q* = 1.5 in all studied crystals. The magnetic field dependence of the activation energy follows a power law of ${U}_{0}(B)\sim {B}^{-\alpha }$ where the exponent *α* is changed from a low value to a high value at a crossover field of *B* = ∼2 T, indicating the transition from collective to plastic pinning in the crystals. Finally, it is suggested that the 3D vortex phase is the dominant phase in the low temperature region as compared to the TAFF region in our series samples.

## Introduction

The efforts to find new superconducting materials have recently led to the discovery of various iron-based superconductors^1–4^. Some of the basic similarities with high-*T*_*c*_ cuprates such as their comparable superconducting transition temperature, a two dimensional layered-crystal structure, the dramatic change of superconducting properties by doping and the proximity to a magnetic transition, etc. have made iron-based superconductors the center of attention in superconductivity researches^1–4^. Therefore, it is very important aspect to compare these two classes of superconductors to uncover the puzzle of high-*T*_c_ superconductivity. However, some important differences are found between these two high-*T*_*c*_ superconducting classes for instance; (1) the parent compound of the iron-based superconductors is not a Mott insulator as in case of the cuprates but a typically bad metal; (2) the iron-based superconductor possesses multiple Fermi surfaces which seems to favor a $\,{s}^{\pm }$-wave pairing symmetry rather than a *d*-wave pairing symmetry as in the cuparates; (3) despite the very high values of upper critical field in both classes, less anisotropic nature of iron-based superconductors make them an ideal candidate in terms of technological applications^1–4^. Furthermore, various attempts have also been made in order to determine that the coupling strength between electrons is induced by the Fermi-surface nesting^5^ or due to proximity of magnetism^6^, however there is no consensus so far.

Among the classes of iron-based superconductors, the 111-type compounds have attracted much attention because even parent compound exhibits superconductivity unlike other classes of iron-based superconductors^7^. For instance, LiFeAs shows bulk superconductivity without doping^8^, however parent NaFeAs compound shows ∼10% superconducting volume fraction and long-range antiferromagnetic (AFM) ordering along with a structural phase transition^7^. The substitution of Co atom for Fe atomic sites in NaFe_1−x_Co_x_As, results in bulk superconductivity and suppresses AFM ordering and the superconducting transition temperature increases up to 23 K at 3% Co doping^7^. Recently, it has been reported that the critical current density in optimally doped NaFe_1−x_Co_x_As (*x* = 0.03) is ∼10^5^ A/m^2^ with a distinct peak effect in magnetic hysteresis which is important in terms of physical and applications point of view^9, 10^. Therefore, it is crucial to understand the vortex dynamics and the reason for the emergence of peak effect in NaFe_1−x_Co_x_As. It is well known that in the mixed state, thermal fluctuations directly affect the vortex motion due to thermally activated flux flow (TAFF) in a superconductor. In the case of iron-based superconductors including “111” compounds, relatively much researches have been conducted for the understanding of the vortex dynamics through TAFF analysis^11–16^. There is a lack of systematic studies on the vortex dynamics in 111-type compounds in terms of chemical doping or applied pressure variations. As a result, the vortex dynamics through TAFF analysis still has important issues to deal with such as understanding of the magnetic field dependence of thermally activated energy, the relationship between the thermally activated energy and coherence length, and to understand the anisotropy of the thermally activated energy between in-plane and out-of-plane in these compounds.

In this paper, in order to solve the aforementioned important issues we systematically study the vortex properties in the under doped (*x* = 0.01), optimally doped (*x* = 0.03), and over-doped (*x* = 0.07) NaFe_1−x_Co_x_As through TAFF resistivity in the mixed state based on electrical resistivity measurements under magnetic fields for *B*//*c*-axis and *B*//*ab*-plane. The magnetic field dependence of the activation energy from the TAFF analysis is well understood as a plastic-flux-creep model due to point defects induced by Co-doping.

## Results and Discussion

**Figure 1:**
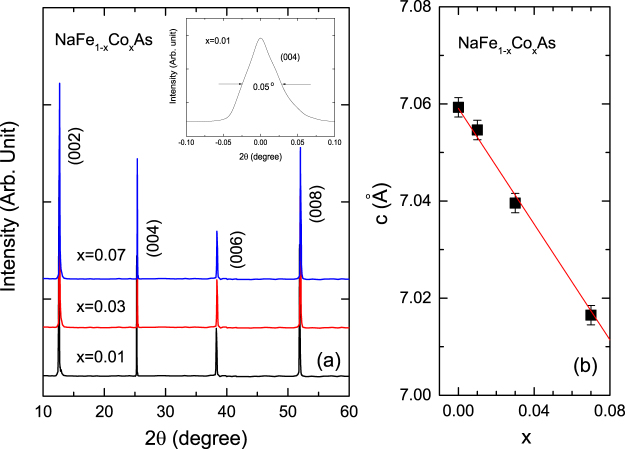
(**a**) XRD patterns for NaFe_1−*x*_Co_*x*_As single crystals. (**b**) The lattice constant of *c*-axis plotted as a function of the Co concentration *x*. The lattice constant of *c*-axis for *x* = 0 is cited from the previous report^17^. Inset in (**a**) is the rocking curve for the (002) deflection of the single crystal for *x* = 0.01.

Figure 1 (a) shows the single crystalline XRD patterns for NaFe_1−*x*_Co_*x*_As (*x* = 0.01, 0.03 and 0.07) single crystals. Only (00 *l*) deflection peaks were recognized and showed a full-width-at-half-maximum (FWHM) of ∼0.05°, indicating that these single crystals are perfectly *c*-axis oriented and of high quality. The Co concentration dependence of lattice constant, *c*, of *c*-axis estimated from the XRD patterns is plotted in Fig. 1(b). The lattice constant *c* for *x* = 0.01 is 7.054 Å, which is consistent with the previous reported values^17, 18^. As shown in Fig. 1(b), the lattice constant decreases linearly with increasing Co concentration indicating the uniform distribution of Co as a dopant in the single crystals, which is again in the agreement with the previous reported results^18^.

**Figure 2:**
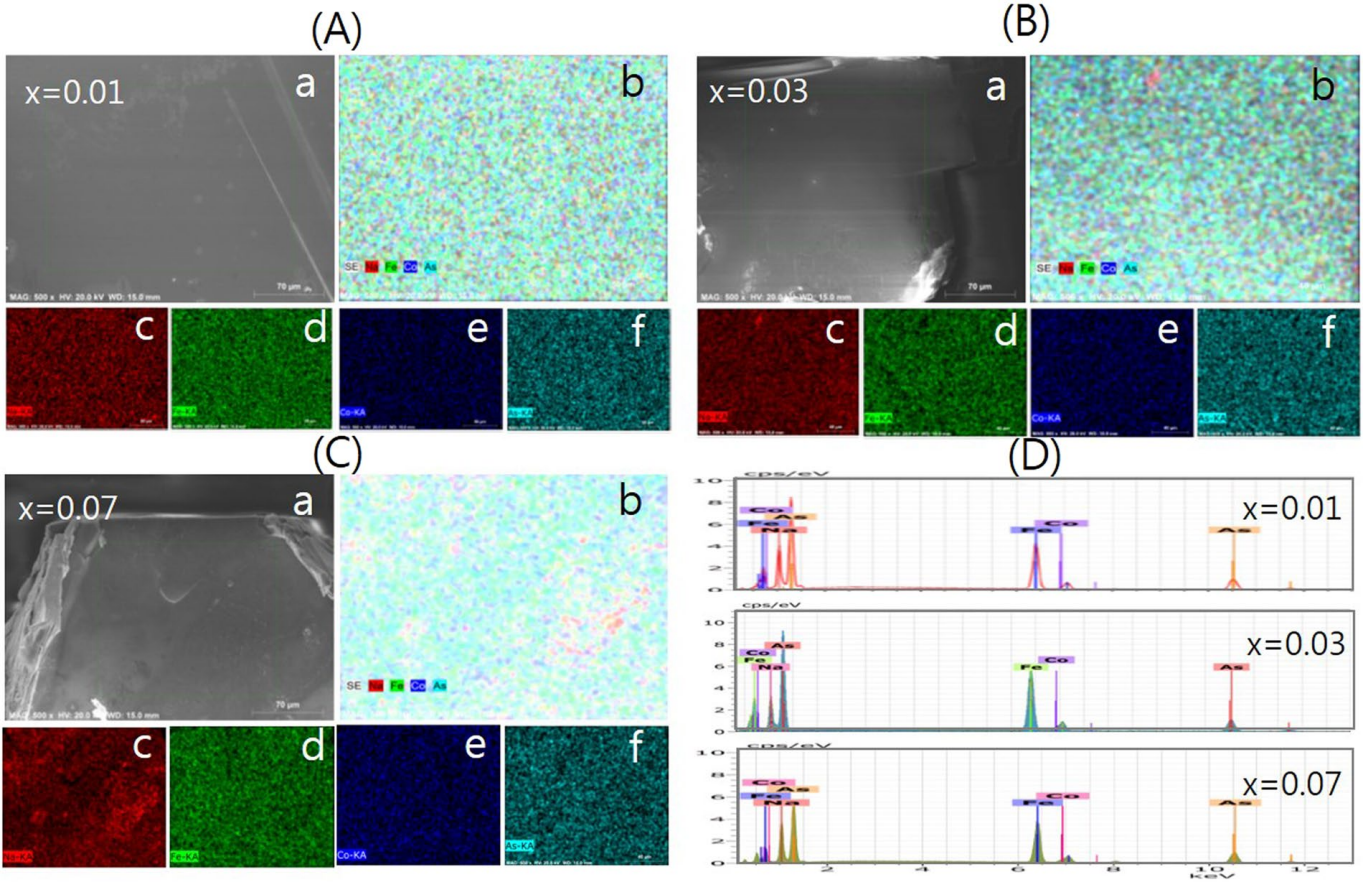
EDS analysis for NaFe_1−*x*_Co_*x*_As single crystals; (**A**) *x* = 0.01, (**B**) *x* = 0.03, and (**C**) *x* = 0.07. (a) SEM image of the single crystal, and (b) the corresponding EDS mapping image for all elements and each separate element (c) Na (d) Fe (e) Co and (f) As. (**D**) EDS spectrum of the NaFe_1−*x*_Co_*x*_As single crystals.

Figure 2 shows the SEM images and the EDS-spectrum for NaFe_1−*x*_Co_*x*_As single crystals. The EDS mappings indicate that each of the Na, Fe, Co and As elements is homogeneously distributed in the samples which is consistent with XRD results. The molar ratios for each element Na, Fe, Co, As in NaFe_1−*x*_Co_*x*_As with *x* = 0.01, 0.03 and 0.07 were listed in Table 1.

**Table 1:** The nominal composition ratio, the composition ratio analyzed by EDS attached in SEM system and the composition ratio analyzed by EDS attached in TEM.

*x* value	Nominal composition ratio of Na:Fe:Co:As	Composition ratio by EDS (SEM) of Na:Fe:Co:As	Composition ratio by EDS (TEM) of Na:Fe:Co:As
*x* = 0.01	1.05: 1.00: 0.00: 1.10	1.02: 0.99: 0.01: 1.00	0.99: 0.99: 0.01: 1.01
*x* = 0.03	1.05: 0.97: 0.03: 1.10	1.01: 0.97: 0.03: 1.00	0.99: 0.97: 0.03: 1.00
x = 0.07	1.05: 0.93: 0.07: 1.10	1.01: 0.93: 0.07: 1.00	0.99: 0.93: 0.07: 1.00

**Figure 3:**
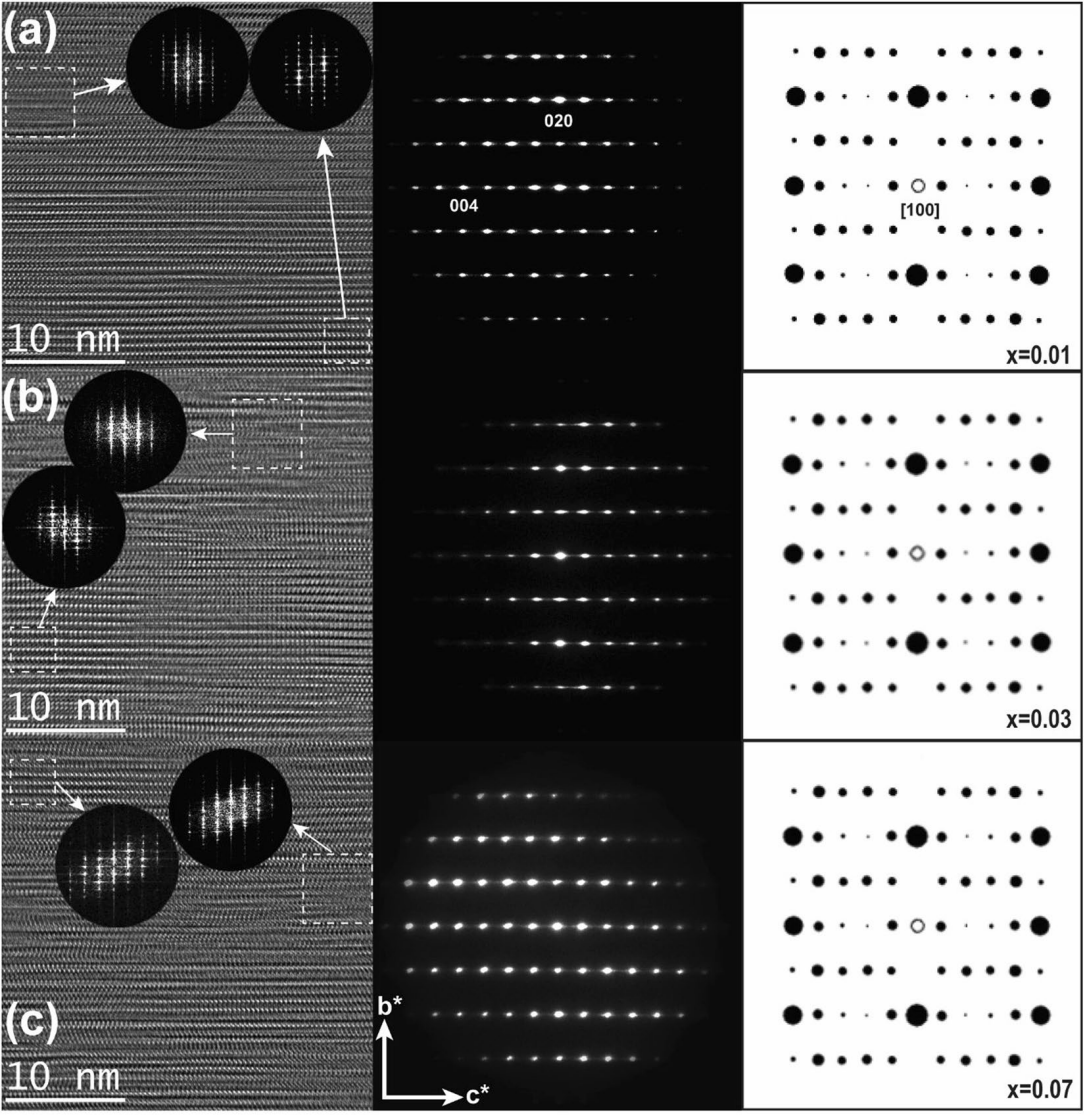
HR-TEM images with attached Fast Fourier transforms (FFT) from the highlighted square areas (left panel) and electron diffraction patterns of NaFe_1−*x*_Co_*x*_As samples along the zone axis [100] at *x* = 0.01 (**a**), *x* = 0.03 (**b**), and *x* = 0.07 (**c**) (middle panel) and theoretical spot diffraction pattern (right panel).

Figure 3 shows HR-TEM images with FFT images, SAED patterns and theoretical spot diffraction patterns for NaFe_1−*x*_Co_*x*_As (*x* = 0, 0.03 and 0.07) single crystals, which provides a crystallographic analysis of the very microscopic area of samples. As shown in the middle panel, the clear SAED patterns consist only of spots that can be seen along the zone axis [100] in the three single crystals indicating that they have good crystallinity. In the left-side panel are the HR-TEM images measured when the single crystals are placed along the zone axis [100] which exhibit some stripes parallel to the planes (001) due to planar defects. The presence of defects can be confirmed by the existence of diffuse 00 l streaks in Fourier transformed images (FTI) of the HR-TEM images contained in the larger square. However, the diffused streaks were hardly observed in FTI obtained from the smaller square. These results indicate that microscopically small single crystals have little planar defects, but single crystals in a larger region where the small single crystals are gathered have the planar defects. However, the planar defect can be caused by an artificial structural defect due to the irradiation of the dual beam FIB and by an intrinsic real structural defect due to Co doping. This former cause has actually been observed in Na_1−y_Fe_2−x_As_2_ samples^19^, which strongly supports that in our samples the formation of the artificial defects can be expected. For instance, if the planar effect is due to the latter reason, the defect concentration should increase as the Co doping increases. However, in our results, the defect concentration was observed almost similarly even with an increase in Co doping. This also supports that the planar defects were artificially formed by the dual beam irradiation.

The SAED patterns are well explained by the theoretical spot diffraction along the zone axis [100]. From fitting the two results, we knew that the values of the lattice parameter *a* were almost unchanged for three samples; *a* = 3.89 Å at *x* = 0.01, *a* = 3.87 Å at *x* = 0.03, and *a* = 3.88 Å at *x* = 0.07, and the values of *c* slightly decreased with increasing Co doping; *c* = 7.06 Å at *x* = 0.01, *c* = 7.05 Å at *x* = 0.03, and *c* = 7.02 Å at *x* = 0.03. The change in the lattice constants due to the Co doping is well consistent with the XRD results, although the lattice constants can be obtained with high accuracy due to the finite spot size in SAED patterns

The chemical composition of NaFe_1−x_Co_x_As samples was analyzed by the EDS analyzer attached with TEM system. This EDS analysis is a more micro-area analysis than the EDS analysis attached in SEM; a few μm for the former and a few hundred μm for the latter. The results of the analysis are listed in Table 1. As shown in the table, the two results are in good agreement. The agreement between the microscopic result and the macroscopic result indicates that the samples are excellent in uniformity.

**Figure 4:**
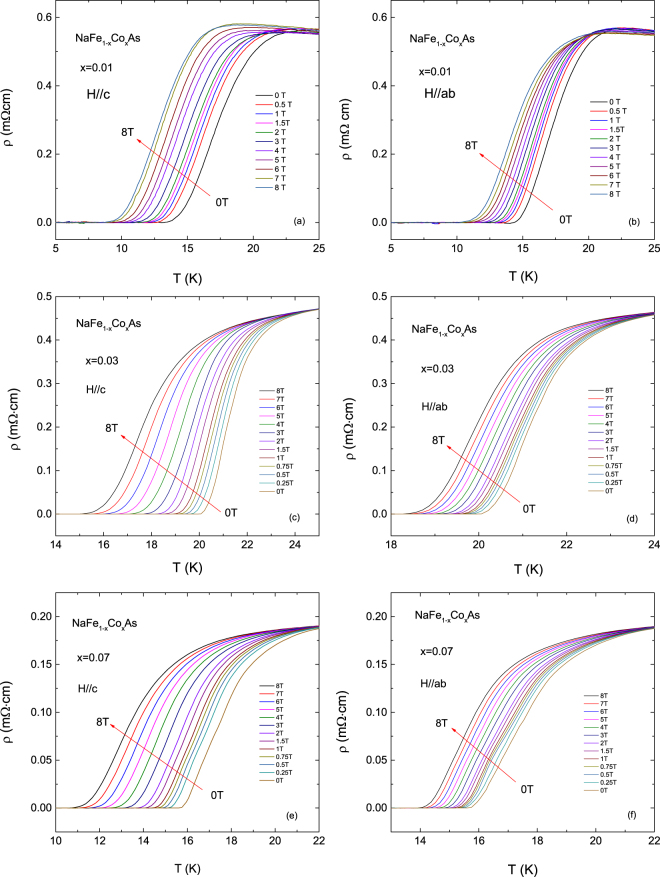
Temperature dependence of the electrical resistivity of NaFe_1−x_Co_x_As single crystals under various applied magnetic fields; (**a**) *x* = 0.01 for *B*//*c*-axis, (**b**) *x* = 0.01 for *B*//*ab*-plane, (**c**) *x* = 0.03 for *B*//*c*-axis, (**d**) *x* = 0.03 for *B*//*ab*-plane, (**e**) *x* = 0.07 for *B*//*c*-axis, and (**f**) *x* = 0.07 for *B*//*ab*-plane.

Figure 4 shows the temperature dependence of the electrical resistivity under various applied magnetic fields for *B*//*c*-axis and *B*//*ab*-plane up to 8 T. In the zero applied magnetic field, the onset (the width) of the superconducting transition is around 19.7 K (4.2 K) for *x* = 0.01, 22.8 K (1.7 K) for *x* = 0.03, and 20.3 K (1.7 K) for *x* = 0.07, respectively. Here the transition width Δ*T*_c_ was estimated as twice the value obtained by the criterion of 10–50% of the normal-state resistivity. Note that the onset transition temperatures are similar to the values reported previously but the Δ*T*_c_ values in our samples are about 20% less than that of the reported values^18^. The sample with *x* = 0.01 exhibits large superconducting transition width compared to other samples. The large transition width is generally known to be due to the sample inhomogeneity, but the sample with *x* = 0.01 does not follow this reason, because the inhomogeneity of the sample was not observed in the detailed macroscopic and microscopic crystallographic analysis discussed above. In NaFeAs, which is expected to have a relatively high homogeneity due to no doping, the superconducting transition was too broad to determine ${T}_{c}^{{\rm{o}}{\rm{n}}}$ accurately^18^. The parent sample and the sample with *x* = 0.01 exhibit an antiferromagnetic order transition at *T*_N_, 41 K for NaFeAs^18^ and 33 K at *x* = 0.01, above *T*_c_, but the samples with *x* = 0.03 and 0.07 do not exhibit the magnetic order. These results suggest that the broad width of the superconducting transition is caused by the intrinsic properties that can occur when superconductivity and magnetic order coexist. Similar phenomenon was observed in Co-doped BaFe_2_As_2_^20^, but it is not as clear as in Co-doped NaFeAs.

The rounding effect due to the thermodynamic fluctuations of the superconducting cooper pairs was distinctly observed around the onset of the transition temperature in both field-directions in each sample. As the magnetic field increases, the onset transition temperature shifts to the lower temperature side with enhanced rounding effect as shown in Fig. 4. However, in all the samples the shift is more pronounced in the case when *B*//*c*-axis than for *B*//*ab*-plane. Furthermore, above the temperature where the electrical resistivity is completely zero, the electrical resistivity showed a distinct tail under magnetic field, which became more evident and extended to lower temperature with increasing magnetic fields. Similar tail has been observed in several layer-structured cuprates and iron-based superconductors and was related with the vortex motion under applied magnetic fields^11–17, 21, 22^.

**Figure 5:**
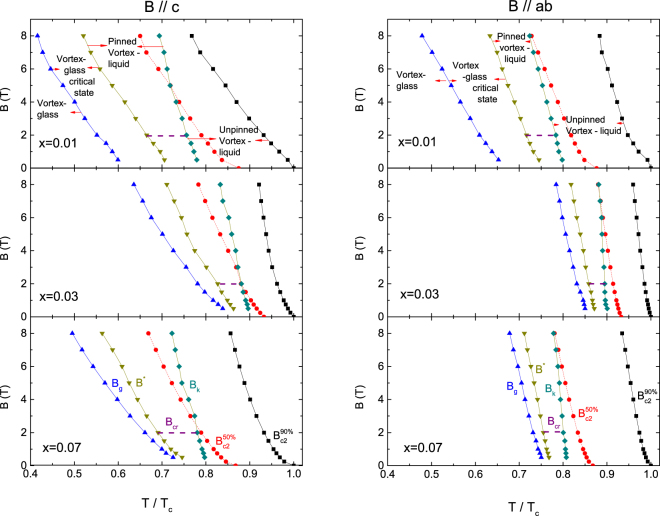
Static phase diagram of NaFe_1−*x*_Co_*x*_As. ${B}_{c2}^{90 \% }\mathrm{and}\,{B}_{c2}^{50 \% }$ are the upper critical fields estimated from 90% and 50% of the normal state resistivity, respectively. The characteristic fields ${B}_{g}(T),\,\,{B}^{\ast }(T)\,$and $\,{B}_{K}(T)$ are determined from the temperature corresponding to the vortex glass-to-liquid transition, the upper-field limit of the critical region associated with vortex glass-to-liquid phase transition and the temperature that separates the vortex liquid phase from the pinned vortex liquid phase, respectively. The crossover field ${B}_{cr}(T)$ is determined from the field dependence of the activation energy.

Figure 5 shows the *B*_*c*2_ − *T* phase diagram determined by the resistivity data shown in Fig. 4, where *B*_c2_(*T*) corresponds to the temperature where the resistivity drops to the 90% and 50% of the normal-state resistivity in the crystals. As shown in Fig. 5, the obtained *B*_*c*2_ is not linear over the entire magnetic field range such that the deviation from linearity is observed above 2 T possibly due to the multiband effect. We estimated the d*B*_*c*2_/d*T* for our crystals in both field directions using the average gradient above 2 T for the data with 50% criterion and listed it up in Table 2. The obtained values for parameters are similar to the values reported previously^23^. Furthermore, the orbital limiting field at *T* = 0 K is given by ${B}_{c2}^{orb}(0)=0.693{T}_{c}{|d{B}_{c2}/dT|}_{{T}_{c}}$ using the Werthamer-Helfand-Hohenberg (WHH) formula within the weak coupling BCS theory^24^ and is listed in Table 2. The ${B}_{c2}^{orb}(0)$ is highest for *x*=0.03 and it is higher for in plane than that of out of plane value. The corresponding Ginzburg-Landau coherence length and the anisotropy ratio defined by ${\rm{\gamma }}\equiv {\xi }_{ab}/{\xi }_{c}$ were obtained and are also listed in Table 2. The coherence length and anisotropy ratio have been already reported for *x* = 0.021 sample;^16^ ${\xi }_{c}={\rm{21.1}}$, ${\xi }_{ab}=$34.4 Å and ${\rm{\gamma }}={\rm{1.63}}$, which lie in between the values of our samples for *x* = 0.01 and 0.03.

**Table 2:** Values of the parameters determined from the resistivity data with 50% criterion for *x* = 0.01, 0.03, and 0.07 for NaFe_1−*x*_Co_*x*_As.

	${{\boldsymbol{|}}{\bf{d}}{{\bf{B}}}_{{\bf{c}}{\bf{2}}}{\boldsymbol{/}}{\bf{d}}{\bf{T}}{\boldsymbol{|}}}_{{\bf{c}}}$ (T/K)	${{\boldsymbol{|}}{\bf{d}}{{\bf{B}}}_{{\bf{c}}{\bf{2}}}{\boldsymbol{/}}{\bf{d}}{\bf{T}}{\boldsymbol{|}}}_{{\bf{a}}{\bf{b}}}$ (T/K)	*T*_*c*_ (K)	*B*_*c*2,*c*_ (T)	*B*_*c*2,*ab*_ (T)	*ξ*_*c*_ (Å)	*ξ*_*ab*_ (Å)	*γ*
*x* = 0.01	2.15	3.41	17.2	25.5	40.5	22.6	35.9	1.6
*x* = 0.03	2.57	7.87	21.3	37.8	115.7	9.6	29.5	3.1
*x* = 0.07	2.68	5.98	17.6	32.5	72.6	14.3	31.8	2.2

In the mixed state of high *T*_c_ superconductor with disorders below *B*_c2_ they induce barriers for the vortex motion and three different situations can be expected; (1) the energy barrier *U*_0_ is lower than the temperature and can be neglected. This state is referred as the unpinned vortex liquid (UVL) state. (2) The energy barrier *U*_0_ is higher than the temperature and plays an important role in vortex motion. This corresponds to the thermally activated flux-flow (TAFF) regime. (3) The barrier grows unlimitedly at low critical current density *j* and the linear resistivity drops to zero. This state is called as the vortex-glass state.

**Figure 6:**
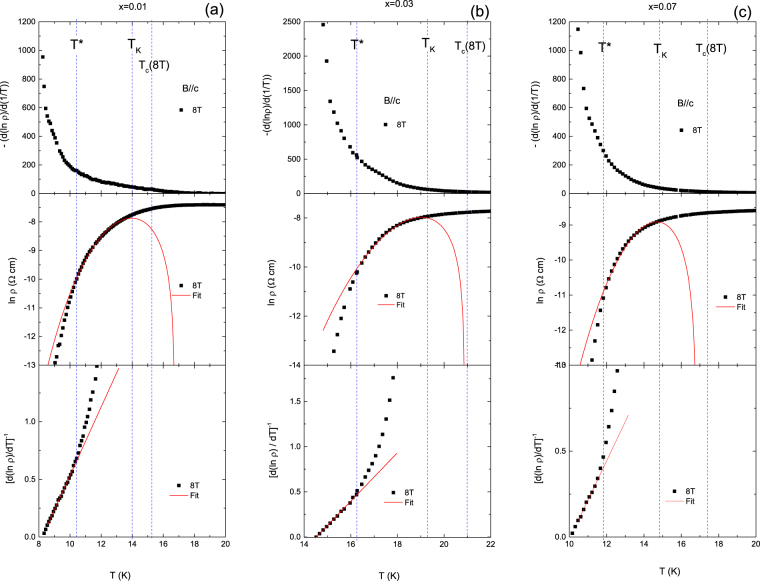
Plots for $-({\rm{d}}\,(\mathrm{ln}\,\rho )/\,{\rm{d}}(1/T))$ vs. *T* (highest panel), $\mathrm{ln}\,\rho$ vs. *T* (middle panel) and ${[{\rm{d}}(\mathrm{ln}\rho )/{\rm{d}}T]}^{-1}$ vs. *T* (lowest panel) at *x* = 0.01 (**a**), 0.03 (**b**) and 0.07 (**c**).

According to the TAFF theory^25^, the resistivity in the TAFF and UVL regimes is defined by

1 $${\rm{\rho }}(T,B)=(2{\rho }_{c}U/T)\exp (-U/{k}_{B}T)={\rho }_{0f}\exp (-U/T),$$

where ${\rho }_{0f}$ is the temperature-independent constant, *U* is the thermal activation energy and *k*_B_ is Boltzmann’s constant. To obtain the activation energy *U*(*T*, *B*) we consider the temperature dependence of the derivative $D=\partial (\mathrm{ln}\,\rho )/\partial ({T}^{-1})$ at various applied magnetic fields and both field directions. The representative results are depicted in the upper panel of Fig. 6. In the Arrhenius relation of $U(T,B)={U}_{0}(B)(1-T/{T}_{c})$, where ${U}_{0}$ is the apparent activation energy and usually plot shows a distinct plateau in the TAFF region. As shown in the figures, however, the plateau is not seen but rather increases as temperature decreases. More specifically, the *D*-value is almost zero above *T*_K_, which is characteristic of the UVL state. In *T* ^*^ < *T* < *T*_K_ expected as TAFF region the *D*-value increases relatively slowly, which indicates that studied samples have a non-linear activated energy of the form of $U(T,\,B)={U}_{0}(B){(1-T/{T}_{c})}^{q}$ ^17, 26^. In *T* < *T* ^*^ the *U*-value increases faster and then diverges, which signifies *U*(*T, B*) as entering into the vortex-glass critical-state region as previously seen in high-*T*_c_ cuprates and FeAs superconductors^11, 26, 27^. Under the non-linear activation energy in the TAFF regime we can derive the equation of the form

2 $$\mathrm{ln}\,\rho =\,\mathrm{ln}\,2{\rho }_{c}{U}_{0}+q\,\mathrm{ln}(1-T/{T}_{c})-\,\mathrm{ln}\,T-{U}_{0}{(1-T/{T}_{c})}^{q}/T.$$

**Figure 7:**
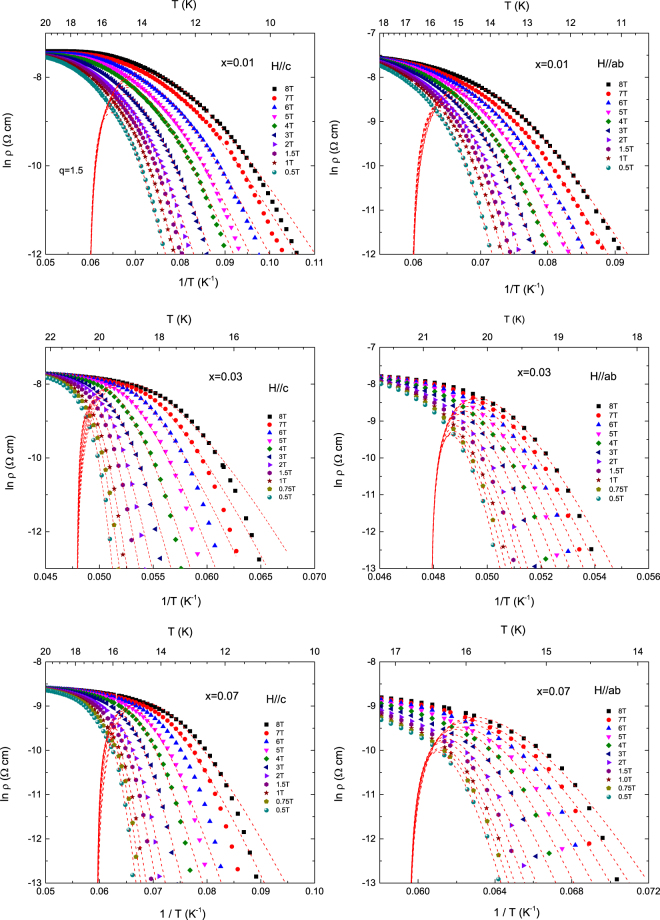
The ln ρ(*T*) versus 1/*T* curves and the curves fitted by the TAFF model by considering the temperature-dependent prefactor and nonlinear relation of $U(T,B)={U}_{0}(B){(1-T/{T}_{c})}^{q}$ at *x* = 0.01 (highest panel), 0.03 (middle panel), and *B*//*c*-axis (lowest panel) under *B*//*c*-axis and *B*//*ab*-plane.

**Figure 8:**
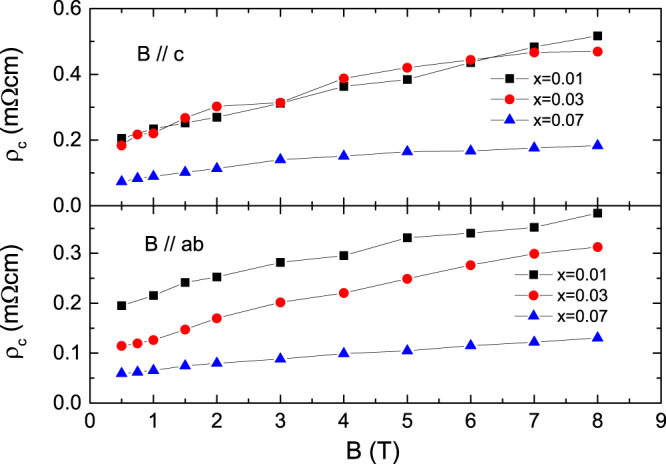
Plot on the field dependence of parameter ${\rho }_{c}(B)$ determined in TAFF analysis.

**Figure 9:**
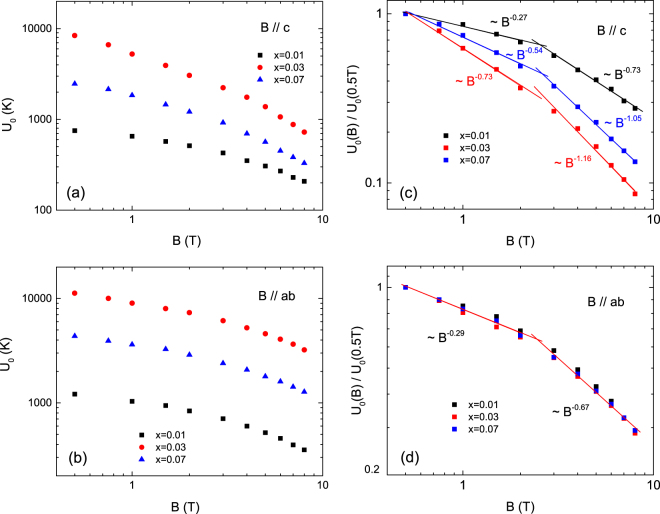
The thermally activated energy ${U}_{0}(B)$ determined by the TAFF model with nonlinear relation of $U(T,\,B)={U}_{0}(B){(1-T/{T}_{c})}^{q}$ for *B*//*c*-axis (**a**) and *B*//*ab*-plane (**b**). The magnetic field dependence of the activation energy normalized by the ${U}_{0}$ at *B* = 0.5 T for *B*//*c*-axis (**c**) and *B*//*ab*-plane (**d**).

To obtain the activation energy we plotted the $\mathrm{ln}\,\rho \,\,$versus 1/*T* data for both magnetic field directions of *B*//*c*-axis and *B*//*ab*-plane at *x* = 0.01, 0.03 and 0.07 in Fig. 7 and tried to fit the data using eq. (2). We found the value of the exponent *q* = 1.5 in both field directions for all the samples and the value of *T*_c_ as 16.8, 20.9 and 16.8 K for *x* = 0.01, 0.03 and 0.07, respectively. The ${\rho }_{c}$ almost linearly increased with applied magnetic fields as shown in Fig. 8. According to the condensation model, *q* = 1.5 is expected in the case of high *T*_c_ superconductors which show 3D vortex behavior, whereas *q* = 2 represents 2D vortex behavior^28–30^. The magnetic field dependence of *U*_0_ evaluated from this fitting is plotted in Fig. 9(a,b) for *B*//*c*-axis and *B*//*ab*-plane for *x* = 0.01, 0.03 and 0.07, respectively. The characteristics of the determined *U*_0_-value are as follows:

1. At *x* = 0.01 (underdoped sample), *U*_0_ decreases from 752 K (1213 K) at *B* = 0.5 T to 207 K (354 K) at *B* = 8 T with increasing applied magnetic field for *B*//*c*-axis (*B*//*ab*-plane). The field dependence of *U*_0_ follows a power law relation [${U}_{0}(B)\sim {B}^{-\alpha }$] for both field-directions. The exponent α has a small value of $0.27$ up to *B* = ∼2 T and a large value ${\rm{\alpha }}=0.73$ over ∼2 T; for *B*//*c*-axis, for *B*//*ab*-plane, ${\rm{\alpha }}=0.29$ when $B < \sim 2\,T$ and ${\rm{\alpha }}=0.70$ when $B > \sim 2\,T$.
2. At *x* = 0.03 (optimally doped sample), *U*_0_ decreases from 8400 K (11250 K) at *B* = 0.5 T to 724 K (3216 K) at *B* = 8 T with increasing magnetic field for *B*//*c*-axis (*B*//*ab*-plane). These values are significantly increased for both field directions as compared with the underdoped sample. The *U*_0_-value is similar to the values reported for SmFeAsO_0.9_F_0.1_ (*T*_c_ = 54 K)^26^ and SmFeAsO_0.85_ (*T*_*c*_ = 50 K)^11^. The power law relation was also observed in the field dependence of the activation energy in case of the optimally doped sample. The exponent significantly increases for *B*//*c*-axis but remains almost unchanged for *B*//*ab*-plane; ${\rm{\alpha }}=0.73$ when $B < \sim 2\,T$ and ${\rm{\alpha }}=1.16$ when $B > \sim 2\,T$ for *B*//*c*-axis and ${\rm{\alpha }}=0.28$ when $B < \sim 2\,T$ and ${\rm{\alpha }}=0.69$ when $B > \sim 2T$ for *B*//*ab*-plane.
3. At *x* = 0.07 (overdoped sample), *U*_0_ decreases from 2470 K (4360 K) at *B* = 0.5 T to 330 K (1277 K) at *B* = 8 T with increasing applied magnetic field for *B*//*c*-axis (*B*//*ab*-plane). These values are between those of the underdoped and optimally doped samples. The field dependence of *U*_0_ in the underdoped sample also follows a power law relation for both directions. Furthermore, the exponents are between those of the underdoped and optimally doped samples for *B*//*c*-axis but is almost unchanged for *B*//*ab*-plane; ${\rm{\alpha }}=0.52$ when $B < \sim 2\,T$ and ${\rm{\alpha }}=1.05$ when $B > \sim 2\,T$ for *B*//*c*-axis and ${\rm{\alpha }}=0.29$ when $B < \sim 2\,T$ and ${\rm{\alpha }}=0.67$ when $B > \sim 2\,T$ for *B*//*ab*-plane.

As mentioned earlier, the activation energy of NaFe_1−*x*_Co_*x*_As follows a power law relation with different value of exponent α near *B* = ∼2 T; in the low magnetic fields α is small, whereas in high magnetic fields α is large similar to iron-based superconductors^12, 13, 31, 32^. Nevertheless, the field dependence of *U*_0_ following a power law has not been well understood so far. Since, as the magnetic field increases, the reduction of *U*_0_ in the form of $\,{U}_{0}(B)\sim {B}^{-\alpha }$ has been described as a result collective elastic creep^33^. However, according to the collective elastic creep theory the activation energy should increase inversely^34–36^.

Very recently, it has been reported that the collective pinning which dominates below *B* = ∼2 T crossover to the plastic pinning for *B* > ∼2 T observed from magnetic properties near *T*_c_/2 in NaFe_1−*x*_Co_*x*_As (*x* = 0.01, 0.03, 0.05 and 0.07) for *B*//*c*-axis^9^. The crossover field shifted to low magnetic field as the temperature increased. As a result of this crossover, the secondary peak was observed in magnetic hysteresis in the optimally doped and overdoped samples but it was absent in the underdoped sample^9^. The peak seems to appear as a crossover from the collective pinning to the plastic pinning, but it was not well-understood about the absence of the secondary peak effect in the underdoped sample.

From this point of view, it is reasonable to assume that the field dependence of *U*_0_ is governed by both the collective pinning and plastic pinning. According to the plastic-flux-creep theory^37–39^, the vortices are plastically deformed and entangled by the weak pinning with point defects, as a result the field dependence of activation energy follows the form *U*_0_ ∼ *B* ^−0.5^. In high magnetic fields, the entangled vortices are cut and disconnected due to the faster motion of vortices relative to each other so the activation energy can be described in the form of *U*_0_ ∼ *B* ^−0.7^ ^27, 37^. The faster reduction in *U*_0_ with magnetic fields was suggested to be due to the entangled vortex liquid behavior in a region with strong pinning by point defects^27^.

Furthermore, by taking into account the magnetic measurement results of NaFe_1−*x*_Co_*x*_As (*x* = 0.01, 0.03 and 0.07)^9^, the crossover field in the temperature range of the TAFF analysis may shift to lower than 2 T. Therefore, it is reasonable to assume that the coexistence of the plastic pinning and collective pinning coexist below 2 T in this temperature range. At *x* = 0.01 for *B*//*c*-axis, in low magnetic field range the exponent is smaller than 0.5, the value expected for the plastic pinning as mentioned above, which is well understood by the coexistence of the collective weak pinning and plastic weak pinning with point defects. However, in the high magnetic fields the exponent is equal to 0.7 in accordance with the plastic weak pinning theory in high magnetic field range. At *x* = 0.03 and 0.07 in *B*//*c*-axis, the both exponents for low and high magnetic fields exceed the expected values which correspond to plastic weak pinning theory as mentioned above, which may indicate the presence of the plastic strong pinning. However, the reason that the exponent in low magnetic field is smaller than that in higher magnetic field may be due to the coexistence of collective pinning and plastic pinning. In case of *x* = 0.07, the plastic pinning becomes more prominent because of lower crossover field^9^ and the exponent in low field region is predicted to be larger, but it is smaller in our result. This indicates that the strength of the pinning for *x* = 0.07 is weaker than that for *x* = 0.03.

In case of *B*//*ab*, three samples show similar magnetic dependence of *U*_0_. In case of *B*//*c*-axis, we considered that point defects in NaFe_1−*x*_Co_*x*_As play an important role in the emergence of plastic pinning which lead to entangled vortex liquid behavior. The entanglement of vortex lines with increasing magnetic field reduces the correlation length along vortex lines which results in to the reduction of the activation energy. It is expected that the smaller the degree of entanglement of the vortex line, weaker the decreasing trend of the activation energy with increasing magnetic field. In NaFe_1−*x*_Co_*x*_As superconductor, Co ions are thought to act as mainly point-pinning centers. Co-ions concentration at *x* = 0.03 and 0.07 increases along the c-axis but is almost unchanged in the ab-plane when compared with the sample with *x* = 0.01, which is determined from the variation of lattice constants. Since, with Co doping, the lattice constants of *a* are almost unchanged but the lattice constants of *c* are linearly decreased with increasing Co^18^. The correlation length of vortex lines for *B*//*ab*-plane is expected to be approximately the same in the three samples and show similar magnetic field dependency regardless of whether the pinning is weak or strong.

**Figure 10:**
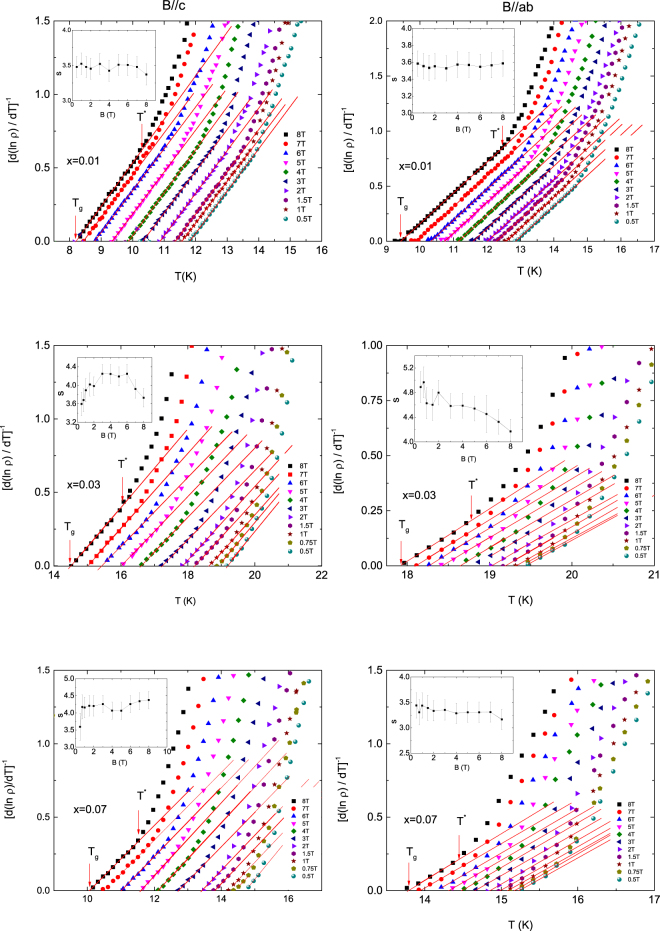
Inverse logarithmic derivative of resistivity for different fields at *x* = 0.01, 0.03 and 0.05. The solid red lines represent fits to the vortex-glass theory using the relation of ${[{\rm{d}}(\mathrm{ln}\rho )/{\rm{d}}T]}^{-1}=(1/s)(T-{T}_{g})$. Insets represent the magnetic field dependence of *s*(*B*).

Analysis of the electrical resistivity below the TAFF temperature region can confirm the presence of the vortex-glass state in NaFe_1−*x*_Co_*x*_As superconductor. The vortex glass theory predicts the temperature dependence of the linear resistivity through the relation ${\rm{\rho }}(T)\propto {(T-{T}_{g})}^{s}$ in the vortex-glass critical region just above the glass transition temperature *T*_g_, where *s* is the critical exponent^40^. As shown in Fig. 10, the linear resistivity below *T* ^*^ is well described by this equation and a straight line is shown in the critical region of the glass transition in a plot of ${[\partial \mathrm{ln}\rho /\partial T]}^{-1}$ versus *T*. The critical exponent *s* is plotted in insets of Fig. 10. The *s*-values for different samples are almost independent of magnetic field in both magnetic field directions and are larger than *s* = 2.7, which is the lower limit of *s* predicted by the 3D vortex-glass picture^33^. The 3D vortices in vortex liquid, which are determined from the *q*-values discussed above, are frozen in to the 3D vortex glass with decreasing temperature, in agreement with the vortex glass theory^40^. The vortex glass transition temperature *T*_g_ determined by the extrapolation is shown in Fig. 5. The vortex glass critical temperature *T* ^*^ drawn in Fig. 5 is defined as the temperature which deviates from the linear electrical resistivity in vortex glass state and is approximately equal to the temperature on the lower side deviating from the TAFF fitting (Fig. 6).

As shown in Fig. 5, the vortex matter phase diagram remarkably depends on the Co-doping concentration. The characteristic fields such as *B*_g_(*T*), *B* ^*^(*T*) and *B*_k_(*T*) shift to low temperature region in a sample with low activation energy and low *T*_c2_. The *B*_k_(T) is the characteristic magnetic field that divides the vortex liquid phase in to a pinned liquid phase and a unpinned liquid phase and is defined as the temperature on the higher side deviating from the TAFF fitting (Fig. 6). This crossover field of *B* ∼ 2 T exists in the magnetic field dependence of the activation energy, which is plotted in the pinned liquid region as *B*_cr_. For *x* = 0.01, the vortex liquid is in a state of coexistence of the weakly collective pinning and the weakly plastic pinning below *B*_cr_ and it is in a state of weakly plastic pinning above *B*_cr_. For *x* = 0.03 and 0.07, the collective pinning and the plastic pinning coexist under the strong pinning limit in *B* < *B*_cr_ but the plastic pinning only exists in *B* > *B*_cr_. From this, it is likely that the peak effect observed in the magnetic hysteresis for *x* = 0.03 and 0.07 occurs when crossing from a collective pinning to a plastic pinning in a strong pinning.

## Conclusions

We investigated the thermally activated flux flow (TAFF) using the magnetoresistivity in single crystals NaFe_1−*x*_Co_*x*_As (*x* = 0.01, 0.03, and 0.07) for *B*//*c*-axis and *B*//*ab*-plane. Results showed that just above the temperature where the electrical resistivity is completely zero, the electrical resistivity shows a distinct tail as a result of thermally activated flux flow which becomes more evident with increasing magnetic fields. This resistivity behavior due to TAFF is well understood by considering the nonlinear temperature relationship of the activation energy. The magnetic field dependence of the estimated activation energy follows a power law of ${U}_{0}(B)\sim {B}^{-\alpha }$ but the exponent increases above the applied magnetic field of *B* = ∼2 T subjected to vortex phase transition. We have found that that in NaFe_1−*x*_Co_*x*_As, crystals, point defects formed as a result of Co doping has played an important role in the vortex pinning properties of our samples. Furthermore, it is suggested that in the underdoped (*x* = 0.01) sample the weak pinning mechanism plays a dominant role in TAFF region whereas in the case of optimally doped (*x* = 0.03) and overdoped (*x* = 0.07) samples the strong pinning mechanism seems to be effective.

## Methods

Single crystals of NaFe_1−x_Co_x_As (*x* = 0.01, 0.03, and 0.07) were grown by the Bridgman method in a vertical vacuum furnace with temperature stability of ±0.5 °C or less^41^. Before growing single crystals, FeAs precursor was prepared by reacting Fe-powder and As-lump at 1050 °C for 120 h. After that, Na: Fe: Co: As were weighed in the molar ratio listed in Table 1 and put it in a BN crucible, and then put all in a molybdenum (Mo) crucible. This all procedure was carried out in a glove box having purified argon (Ar) gas atmosphere with the level of H_2_O and O_2_ contents is 0.1 ppm or less. The Mo crucible was arc welded in Ar gas atmosphere to avoid the escape of highly volatile As and Na elements. In the final stage, the sealed Mo crucible was heated up to 1350 °C at a rate of 60 °C/h and kept for 72 hrs in a vertical vacuum electric furnace composed of tungsten mesh, after that the crucible was slowly moved to the bottom of the heater at a rate 1.7 mm/h. After the completion of the heat treatment, a number of single crystals of size 5 × 5 × 1 mm^3^ were obtained.

In order to analyze the crystalline structure and lattice constant, x-ray diffraction (XRD) for a cleaved surface of single crystals was performed with a Cu-K_α_ radiation source. The detailed analysis of the compositions of the single crystals was performed using an energy-dispersive x-ray spectroscopy (EDS) and mapping method. The EDS mappings were measured by a Hitachi S-4800 scanning electron microscope with an energy dispersive x-ray analyzer (Bruker QUANTAX). The accelerating voltage and the applied current were 20 kV and 10 μA. The EDS mappings were performed for each of the Na, Fe, Co and As elements.

For the crystallographic analysis of the microscopic part of samples, the high resolution-transmission electron microscopy (HR-TEM) pattern and selected area electron diffraction (SAED) pattern were measured using a Hitachi HF-3300 at a 300 kV accelerating voltage. Spot diffraction patterns were theoretically calculated using the non-commercial software (CrysTbox)^42^. In order to measure HR-TEM, we made the NaFe_1−x_Co_x_As samples thinner than 100 nm from the ab plane using a dual-beam FIB with accelerating voltage of 40 kV and then we quickly transferred them to the TEM instrument in order to prevent their oxidation. All images were acquired with the program of GMS3 (Gatan) and the average background subtraction filtering (ABSF) was used to obtain better contrast TEM images. Quantitative analysis of EDS was also performed using a Bruker QUANTAX attached to TEM.

The electrical resistivity measurements of series single crystals were carried out by using a 9 T Physical Property Measurement System (PPMS, Quantum Design, Inc.). Since, the samples are highly sensitive to air and moisture, therefore all the work such as sample cutting and lead-wires connection was performed in the glove box mentioned above. The transversal resistivity was measured down to 2 K using a standard four-probe method at a current density of ∼1 A/cm^2^. The applied magnetic fields of *B* = 0, 0.5, 0.75, 1, 1.5, 2, 3, 4, 5, 6, 7 and 8 T were used for both field directions as *B*//*c*-axis and *B*//*ab*-plane for measuring the magnetoresistivity. The samples used in this study were taken from the same batches that were used in our recent study on magnetic properties and specific heat results^9^.
